# "Idiopathic" Hæmatoporphyrinuria

**Published:** 1909-08-07

**Authors:** 


### "IDIOPATHIC" HÆMATOPORPHYRINURIA.

It is well enough known that Hæmatoporphyrinuria is one of the grave symptoms that may result from the administration to patients of drugs such as trional, sulphonal, or tetronal. It is less well known, perhaps, that there are other patients in whom a precisely similar condition may arise without any apparent cause. These seemingly "idiopathic" cases are possibly associated with some decided anomaly in the hepatic functions. Whatever their pathology, they are serious enough; for although some recover, and others survive for a long time with recurrences of the complaint at intervals, some prove rapidly fatal, as in the following instance recorded by Dr. Parsons in Ireland.

The patient came under observation early one June, suffering from diffuse abdominal pain, vomiting, and constipation. The urine, on standing, became of a deep port-wine colour, but although intense hæmaturia at first suggested itself, neither albumen nor blood was present. Spectroscopically, hæmatoporphyrin was detected; although, as in almost all these cases, the colouration of the urine was only in part due to this, some other pigment, as yet unidentified, being present in far larger quantities than the hæmatoporphyrin itself. Neither sulphonal nor trional had been administered, and no cause for the hæmatoporphyrinuria could be made out. After ten days the patient's condition was so much improved that she was sent away for a change of air. The trouble recurred, however, almost at once, and she was again seen at the end of June. Abdominal pain and sleeplessness were prominent symptoms, and the urine again developed the same colour as it had done in the first attack.

Towards the end of the first week after re-admission to hospital the patient complained of severe pain in her feet. On July 7 her arms were found to be almost powerless, and the woman was so ill and feeble that she could barely move in bed. On July 10 incontinence of fæces developed. A few days later, swallowing became difficult, and death took place in a little over two weeks from the re-admission to hospital and in less than seven weeks from the first onset of symptoms.

In addition to its port-wine colour, another remarkable feature of the urine was that it showed little or no tendency to decompose, even when kept for more than a year.

